# No luck replicating the immune response in twins

**DOI:** 10.1186/s13073-015-0156-0

**Published:** 2015-03-25

**Authors:** Alan G Baxter, Philip D Hodgkin

**Affiliations:** Comparative Genomics Centre, Molecular Sciences Bldg 21, James Cook University, Townsville, Queensland 4811 Australia; Immunolology Division, The Walter and Eliza Hall Institute of Medical Research, 1G Royal Parade, Parkville, Victoria 3052 Australia; Department of Medical Biology, The University of Melbourne, Parkville, Victoria 3010 Australia

## Abstract

Recent twin studies highlight the astonishing impact of non-heritable contributions to our immune health and wellbeing. Immunologists, long familiar with heterogeneity generated from within cells, must now grapple with heterogeneity between and within individuals which is present to an extraordinary degree. The capacity to interpret and find patterns in the face of such immune system diversity may be limited when sampling is restricted to blood, necessitating the development of new approaches.

## Immune system heterogeneity

It is a point of some embarrassment to immunologists (and disappointment to medical insurers) that patients cannot walk into a doctor’s surgery or pathology clinic and receive a test to determine the health of their immune systems. This is not for lack of things to measure; indeed, the immune system, many components of which are carried in peripheral blood, offers hundreds of cell types and perhaps thousands of molecules that can be monitored. Our inability to offer this service results from a problem more fundamental than technological failure. At its core, the issue is the immense heterogeneity and variability in immune cells and proteins.

A study recently published in *Cell* by Brodin *et al*. [1] attempted to determine the proportion of the variance seen in immunological traits within a population that is contributed by heritable factors (heritability). They measured the levels of 51 secreted factors (such as the cytokines interleukin (IL)4, IL7 and interferon gamma) and the frequencies of 95 immune cell subsets (for example, T cells, B cells and neutrophils) in peripheral blood, as well as 58 cellular responses to cytokine stimulation, in 78 monozygotic (identical) and 27 dizygotic (fraternal) twin pairs. Even monozygotic twins differed greatly from each other in most of the assays performed, and the authors concluded that in almost 80% of the measurements, heritable factors had only a minor role, and in almost 60%, the contribution of these factors was so low as to be undetectable.

Peter Medawar made the point that the choice of reference population is critical in assessing heritability [2]. For example, in a hypothetical community in which defects in phenylalanine hydroxylase were universal and the use of phenylalanine rare, phenylketonuria would effectively only exist in cases of poisoning and have low heritability. In contrast, in communities in which phenylalanine hydroxylase deficiency is rare and phenylalanine is widely used as the artificial sweetener aspartame, phenylketonuria occurs in almost all people bearing the deficiency and the disease has high heritability. In the case of Brodin’s twin study, the subjects were healthy adults from metropolitan Palo Alto, California, in the United States. People with the known causes of heritable immune deficiency were excluded, as were those with a wide range of autoimmune diseases that together affect perhaps 5% of the population. Nevertheless, taking the data at face value, it is surprising that the heritability of so many immune measurements is so low, when the heritability of immune diseases is relatively high. For example, the heritability of type 1 diabetes, lupus and autoimmune hypothyroidism is around 80% [3-5]. Similarly, studies of responses to numerous childhood vaccines are highly correlated within twins [6]. In contrast, Brodin *et al*. [1], who were looking mainly in adults and were measuring antibody titers in response to seasonal flu vaccination, found no evidence of heritability.

## Explaining the discrepancy

So what is going on? How can immune disease be highly heritable while measurable immune parameters are so disparate?

Many accessible immune markers not only show heterogeneity between individuals, but also vary widely over time in a single person. The normal ranges cited for leukocyte subsets by clinical laboratories vary fourfold for most lineages, and up to tenfold for monocytes. Longitudinal studies of some cell types show either high variation over time (for example, T regulatory cells [7]), or very little (for example, B-cell subsets [8]). Brodin *et al*. [1] examined a single time point for each subject, raising the question of how different twins within a pair are from each other, compared to testing the same twin twice. To address this issue in part, Brodin *et al*. compared variation in multiple samples collected from individual donors over time. Of the 182 assays tested repeatedly, for only 26 did 95% of the replicate measurements fall within a third of the mean value.

The second possible explanation for the observed lack of heritability is that the immune system is complex and immune function is an emerging phenomenon resulting from networks of interactions that are summated in ways we do not yet fully understand. One of the compelling conclusions of the Brodin study is that this already complex system changes further with time through the cumulative action of non-heritable influences. What are these influences? A strong case is made that infection is a major effector, as chronic cytomegalovirus (CMV) exposure significantly altered the phenotypes of infected versus non-infected twins. However, the changes may also arise from stochastic processes. T- and B-cell antigen receptors arise from probabilistic gene assortment and differ between identical individuals, affecting their immune responses [9]. Other stochastic fate decisions recently identified for T- and B-cell responses are also likely to be magnified in influence over time, especially if responses are channeled through limiting numbers of responding cells [10].

Could these time-dependent, non-heritable changes, as reflected in blood, serve as diagnostic indicators or predictors of immune status? Perhaps, yes. The highly variable pre-immune patterns of naïve and memory cell types were successfully correlated and used to predict vaccine outcome in another study, by Tsang *et al*. [8].

The third possible explanation for the high heritability seen with immune disease, but low heritability for immune blood tests, is almost trivial. The blood is not a significant site of immune activity: the complex interactions that take place between cells and cytokines occur in lymphoid organs and in the tissues. With the exception of the acute phase response, circulating titers of most cytokines may have limited functional impact. Similarly, for most lymphocytes, blood is a vehicle of recirculation, rather than a site of activation and effector function. It is possible that we will have to accept that the blood will always be a poor window into our immune corpus, despite our recent ability to measure its constitution in many dimensions.

## Wider implications

Where does this leave the dream for an immune system ‘fitness check’? Blood tests for several immune parameters are already capable of detecting well-characterized immune diseases; allergies, diabetes, lupus and immunoglobulin deficiencies can all be diagnosed by blood tests. In contrast, significant questions remain over the importance of differences in results within normal ranges in otherwise healthy people. The Brodin *et al.* study highlights the considerable variation accumulated over a lifetime in many peripheral blood immune parameters. Whether this variation is necessarily informative, or even meaningful, is not yet clear. It is clear, however, that the implications of this discovery are challenging. Monitoring the health and potential of our changing immune systems is not going to be easy, and may never be accurate.
